# Synergistic Antibacterial Effects of Chitosan-Caffeic Acid Conjugate against Antibiotic-Resistant Acne-Related Bacteria

**DOI:** 10.3390/md15060167

**Published:** 2017-06-08

**Authors:** Ji-Hoon Kim, Daeung Yu, Sung-Hwan Eom, Song-Hee Kim, Junghwan Oh, Won-Kyo Jung, Young-Mog Kim

**Affiliations:** 1 Department of Food Science and Technology, Pukyong National University, Busan 48547, Korea; kjhy1126@naver.com (J.-H.K.); ksonghee93@gmail.com (S.-H.K.); 2 Department of Chemical and Biological Engineering, University of Saskatchewan, Saskatoon, SK S7N 5C5, Canada; day852@mail.usask.ca; 3 Korea Food Research Institute, Sungnam 13539, Korea; dalgoo52@gmail.com; 4 Department of Biomedical Engineering, Pukyong National University, Busan 48513, Korea; jungoh@pknu.ac.kr (J.O.); wkjung@pknu.ac.kr (W.-K.J.); 5 Marine-Integrated Bionics Research Center, Pukyong National University, Busan 48513, Korea

**Keywords:** acne vulgaris, antibiotic resistance, chitosan-phytochemical conjugates, synergistic antibacterial effect

## Abstract

The object of this study was to discover an alternative therapeutic agent with fewer side effects against acne vulgaris, one of the most common skin diseases. Acne vulgaris is often associated with acne-related bacteria such as *Propionibacterium acnes*, *Staphylococcus epidermidis*, *Staphylococcus aureus*, and *Pseudomonas aeruginosa*. Some of these bacteria exhibit a resistance against commercial antibiotics that have been used in the treatment of acne vulgaris (tetracycline, erythromycin, and lincomycin). In the current study, we tested in vitro antibacterial effect of chitosan-phytochemical conjugates on acne-related bacteria. Three chitosan-phytochemical conjugates used in this study exhibited stronger antibacterial activity than that of chitosan (unmodified control). Chitosan-caffeic acid conjugate (CCA) showed the highest antibacterial effect on acne-related bacteria along with minimum inhibitory concentration (MIC; 8 to 256 μg/mL). Additionally, the MIC values of antibiotics against antibiotic-resistant *P. acnes* and *P. aeruginosa* strains were dramatically reduced in combination with CCA, suggesting that CCA would restore the antibacterial activity of the antibiotics. The analysis of fractional inhibitory concentration (FIC) indices clearly revealed a synergistic antibacterial effect of CCA with antibiotics. Thus, the median sum of FIC (∑FIC) values against the antibiotic-resistant bacterial strains ranged from 0.375 to 0.533 in the combination mode of CCA and antibiotics. The results of the present study suggested a potential possibility of chitosan-phytochemical conjugates in the control of infections related to acne vulgaris.

## 1. Introduction

Acne vulgaris is one of the most commonly observed skin diseases, affecting young adults (11–30 years). It can cause permanent physical scarring resulting in intense emotional scars, which might lead to clinical depression and social phobias [1,2]. *Propionibacterium acnes*, *Staphylococcus epidermidis*, and *S. aureus* are known skin pathogenic bacteria associated with acne vulgaris. These bacteria are related to the development of inflammation and abnormal follicular keratinization [3]. *P. acnes* develops inflammatory acne by chemotactically-attracted neutrophils by metabolizing sebaceous triglycerides into fatty acids [4,5]. Normally, antibiotics are used as acne treatment to kill the bacteria. Among them, erythromycin, lincomycin, and tetracycline are usually chosen for the antibiotic therapy [6,7]. However, long-term usage of antibiotics is associated with complications, such as outbreaks of resistant bacteria, immune hypersensitivity, and organ damage [5,8]. Therefore, developing alternative therapeutic agents with fewer side effects and strong antibacterial effect need to be researched.

To overcome the problem of side effects, several candidate substrates, such as traditional Chinese medicines, essential oils, herbs, and chitosan, have been investigated for the treatment of acne [9,10]. Among several candidate substrates, chitosan is an especially attractive resource to develop marine-derived antimicrobial agents due to their low cost and suitability for mass production [11,12]. Chitosan is one of mucopolysaccharides of marine origin, being biodegradable, biocompatible and having low toxicity. Chitosan has been used in the pharmaceutical industry due to its anti-inflammatory, antioxidant, and enzyme-inhibitory properties [13,14,15]. Furthermore, chitosan offers the advantage of easy chemical modifications on account of the primary amino group at the 2-position of each polymer subunit with other biological activities [16]. Therefore, the development of novel chitosan derivatives with functional properties is increasingly in demand [17]. However, as described above, most studies related to chitosan and its derivatives have been focused on the biological activities, such as antioxidant, antitumor, and anti-inflammatory effects, not antimicrobial activity [18,19,20,21]. Our group previously developed chitosan derivatives conjugated with phytochemicals (caffeic acid, ferulic acid, and sinapic acid), which exhibited strong antibacterial activity against pathogenic bacteria [14,21]. Thus, as part of our ongoing investigation on chitosan-conjugated derivatives for the treatment of acne vulgaris, the present investigation reports the antimicrobial activity of chitosan derivatives conjugated against *P. acnes*. The results of this study will provide valuable information on the development of an alternative acne vulgaris therapeutic agent.

## 2. Results

### 2.1. Antibacterial Effect of Chitosan-Phytochemical Conjugates against Acne-Related Bacteria

Hydroxycinnamic acids (HAs) (having sinapic acid, caffeic acid, and ferulic acid) are naturally occurring phytochemicals from plants and have been used in pharmaceuticals, cosmetics, and the food industry [21,22,23]. In this study, chitosan-phytochemical conjugates were synthesized by conjugating HAs with chitosan following the previous method [17,24]. It was then assessed for the antibacterial activity of chitosan-phytochemical conjugates against acne-related bacteria. According to Lee et al. [25], Chitosonic^®^ acid can be used as a potent antimicrobial agent against Gram-positive bacteria, Gram-negative bacteria, and fungi. However, they did not employ the most common antibacterial method such as the tube dilution method and agar dilution methods. In order to determine its antibacterial effect of chitosan-phytochemical conjugates quantitatively, the antibacterial activity of the chitosan-phytochemical conjugates against acne-related bacteria was investigated using an MIC assay. The MIC values of the chitosan-phytochemical conjugates were evaluated by the two-fold serial dilution method, and the results are given in Table 1. The MIC of the unconjugated chitosan was in the range of 16–512 μg/mL against acne-related bacteria. Moreover, the chitosan-phytochemical conjugates had lower MIC values than these of the unconjugated chitosan ranging from 8 to 256 μg/mL. The highest antibacterial activity was observed in the treatment of CCA. The MICs of CCA were determined in a range of 8–256 μg/mL against acne-related bacteria tested in this study. CFA and CSA showed MIC values of 16–256 μg/mL against acne-related bacteria. 

The antimicrobial activities of essential oils, medicinal plants, and chemicals against *P. acnes* have been reported. Zu et al. [10] previously reported that the MICs of the essential oils from thyme, cinnamon, and rose against *P. acnes* were 0.0016% (*v*/*v*), 0.0016% (*v*/*v*), and 0.031% (*v*/*v*). The duzhong extract showed the greatest antimicrobial activity against *P. acnes* with an MIC of 0.5 mg/mL and the yerba mate extract showed moderate antibacterial activity against *P. acnes* with an MIC of 1.0 mg/mL [26]. Although the antimicrobial activity of the chitosan-phytochemical conjugate against methicillin-resistant *S. aureus* (MRSA), foodborne pathogens, and fish pathogens are reported, a limited number of studies have been performed to assess the antimicrobial activity of chitosan-phytochemical conjugates against *P. acnes* [21,27,28]. 

### 2.2. Antibiotic Resistance of Acne-Related Bacteria against Commercial Antibiotics

The generally available therapeutic option for the treatment of acne vulgaris is an antibiotic application to kill the acne-related bacteria. Tetracycline, erythromycin, and lincomycin are the primary choice of antibiotics in the treatment of acne-related bacterial infections [6,7]. With the increase in antibiotic application, the increased prevalence of antibiotic-resistant bacteria has been reported [29]. Additionally, the antibiotic resistance of acne-related bacteria has been previously reported. According to the report by Lee et al. [30], *P. acnes* is highly resistant to erythromycin (an MIC of 2048 μg/mL). However, *S. epidermidis* and *S. aureus* were sensitive to erythromycin, tetracycline, and lincomycin in the MIC ranges of 0.125 μg/mL to 8 μg/mL. In addition, Oprica et al. [31] reported that *P. acnes* has a high resistance to erythromycin and clindamycin. The antibiotic resistance patterns of acne-related pathogens used in the present study were qualitatively determined by an MIC assay (Table 2). MIC breakpoints can assist in determining if an antibacterial is potentially useful in the treatment of a bacterial infection. To compare the antibiotic resistance of acne-related bacteria against commercial antibiotics, the antibiotic-resistant profile of each bacteria was then determined based on the analysis of the MIC breakpoint [32].

### 2.3. Synergistic Antibacterial Effect between CCA and Antibiotics against Acne-Related Bacteria

With the emergence of multidrug-resistant bacteria, the need for new antibiotics or therapeutic agents has increased [33,34]. One of the effective strategies in developing alternative therapies is restoring antibiotic efficacy with non-antibiotic natural products against drug-resistant bacteria [14,35,36,37]. Based on these reports, an interaction between chitosan-phytochemicals and commercial antibiotics were estimated by the checkerboard method, as stated above, and the results are presented in Table 3. Among the chitosan-phytochemicals, CCA presented the highest antibacterial activity against acne-related bacteria and, hence, CCA was chosen for further studies.

As shown in Table 2, the MICs of tetracycline, erythromycin, and lincomycin against antibiotic-resistant *P. acnes* strains ranged from 16 to 1024 µg/mL. However, the MICs against the *P. acnes* strains were dramatically decreased in combination with CCA (Table 3). The MICs of tetracycline against *P. acnes* KCTC 3314 and isolate 2874 strains were fairly reduced, up to 4 μg/mL when applied in combination with 256 μg/mL of CCA. Comparing the MICs of tetracycline alone (16 to 32 μg/mL), the MICs decreased two- to three-fold in the combination of tetracycline-CCA. In addition, the MICs of erythromycin and lincomycin against *P. acnes* strains were also dramatically reduced two- to four-fold in combination with CCA. As a result, the median FIC indices were from 0.502 to 0.504. Thus, these results indicated that the combination of CCA with commercial antibiotics used in acne infection resulted in a synergistic effect against the antibiotic-resistant *P. acnes* strains. In analogy to the antibacterial effect against the antibiotic-resistant *P. acnes* strains, a synergistic antibacterial effect against an antibiotic-resistant *P. aeruginosa* KCTC 1637 strain was also observed in CCA-erythromycin and CCA-lincomycin combinations. The median ∑FIC indices were in the range of 0.375–0.500, indicating a marked synergy effect between lincomycin and CCA and a weak synergy between erythromycin and CCA. These results were also in agreement with the research of Eom et al. [14] that chitosan-derived conjugates clearly reversed the antibacterial activity of β-lactam antibiotics against MRSA in the combination mode. The median ΣFIC indices were in the ranges of 0.375–0.563. Additionally, it has been previously reported that phlorotannins of an edible brown seaweed *Eisenia bicyclis* exhibited a synergistic antibacterial effect with the FIC indices ranging from 0.502 to 1.000 against antibiotic-resistant *P. acnes* strains in combination with the antibiotics used in this study [5]. Kim et al. [38] reported a synergistic effect between an edible brown algae (*Sargassum serratifolium*) extract and the antibiotics, with the median ∑FIC indices from 0.270 to 0.550 against *P. acnes* strains. Compared with these results, it was clear that CCA in combination with the antibiotics showed comparably strong synergistic antibacterial effect against antibiotic-resistant acne-related bacteria.

## 3. Discussion

In this research, we determined the antibacterial effect of chitosan-phytochemical conjugates on acne-related bacteria, such as *P. acnes*, *S. epidermidis*, *S. aureus*, and *P. aeruginosa*. The results of the antibacterial effect of chitosan-phytochemical conjugates against acne-related bacteria were in accordance with the previous reports that the chitosan-phytochemical conjugates exhibited higher antimicrobial effects than that of unconjugated chitosan [14,21]. The MICs of chitosan-phytochemical conjugates were in the range of 8 to 512 μg/mL against acne-related bacteria. Meanwhile, the MICs of the unmodified chitosan ranged from 128 to 1024 μg/mL. Thus, the chitosan-phytochemical conjugates possessed lower MIC values than those of the unconjugated chitosan, indicating that the conjugation increased antibacterial activity. As shown in Table 1, the antimicrobial efficacy of chitosan-phytochemical conjugates was proved to be strain dependent. Chitosan-phytochemical conjugates had higher antibacterial activity against *S. aureus*, *S. epidermis*, and *P. aeruginosa* than *P. acnes*. This may be due to the difference in the modes of antibacterial activity. Chitosan-phytochemical conjugates may increase the osmotic pressure-induced disruption and shrinkage of the bacterial membrane because of a reduction in the permeability of the membrane to intracellular components in *S. aureus*, *S, epidermis*, and *P. aeruginosa* [18,35]. In *P. acnes*, chitosan-phytochemical conjugates may operate via multiple mechanisms [39]. Like other studies proposed, the chitosan-phytochemical conjugates may also form a barrier on the bacterial surface and prevents the entry of nutrients [40]. Interestingly, chitosan and the chitosan-phytochemical conjugates showed very strong antibacterial activity against *P. aeruginosa* in the MIC range of 16 to 32 μg/mL, while Mazurova et al. [24] reported that MIC values of natural substances (gallic acid, methyl gallate, ethyl gallate, propyl gallate, octyl gallate, carvacrol, thymol, and eugenol) ranged from 300 to 2400 μg/mL. The mode of antibacterial activity is a complicated process that differs between Gram-positive and Gram-negative bacteria due to different cell surface characteristics. In several studies, stronger antibacterial activity was apparent against Gram-positive and Gram-negative bacteria [41]. Generally, *P. aeruginosa* has high-level resistance to antibiotics and substances derived from natural materials due to the low permeability and the action of multidrug efflux pumps [5,42]. Bacterial cell surface is a unique structure and a major target for the development of antibacterial agents. Positively charged chitosan and negatively charged bacterial cell surfaces interact with each other, leading to weakness of the cell wall accompanied by either cell lysis or cell wall damage alone [30,42]. In analogy to chitosan-polyphenol conjugates, the antibacterial activity of chitosan-phytochemical conjugates were accelerated by affecting the integrity of bacterial cell envelope and altering cell permeability resulting from interaction with the envelope [30,43]. To date, there have been several reports on the control of acne-related bacteria. However, these studies focused on the antibacterial activity of natural compounds derived from terrestrial organisms [31,32,33]. Furthermore, these compounds exhibited lower antibacterial activity against acne-related bacteria compared to the chitosan and chitosan-phytochemical conjugates [3,44]. Thus, to the best of our knowledge, this is the first study to show chitosan-phytochemical conjugates exhibiting antibacterial activity against acne-related bacteria. 

In particular, both *P. acnes* strains have high-level resistant to erythromycin and lincomycin with MICs of 1024 μg/mL (Table 2). However, *P. acnes* isolate 2875 strain is an antibiotic susceptible bacterium to the three antibiotics. In addition, previously, we also reported the safety of chitosan conjugates in human keratinocyte cell lines [45]. Considering these points, chitosan and its phytochemical derivatives with broad spectrum antimicrobial activity are associated as being safe to animal cell lines and will be very useful for commercial exploitation in the pharmaceutical industry.

Among chitosan-conjugated derivatives, CCA showed the highest antibacterial activity and also exhibited the synergistic antibacterial effect in combination with tetracycline, erythromycin, and lincomycin against acne-related bacteria. Resistance to erythromycin, tetracycline, and lincomycin in *P. acnes* has primarily been related to target site mutations in the genes encoding the 23S rRNA, the 16S rRNA, and *erm(X)* [46]. The restored antibacterial activities with CCA and antibiotics against acne-related bacteria may help prevent mutations in the 23S rRNA gene, 16S rRNA, and *erm(X)*. Although further experiments are needed to clarify the detailed mechanisms of the synergistic effect between CCA and commercial antibiotics to treat acne vulgaris, synergistic antibacterial effects of CCA suggest that CCA may restore the antibacterial activity of old commercial antibiotics, which had lost their antibacterial activity against some antibiotic-resistant bacteria. In this study, the chitosan-phytochemical conjugate might potentially be used as the alternative treatment of the antibiotic-resistant bacteria. However, some issues still remain to be examined in future studies. For example, an important issue is to explain the restoring mechanism of antibacterial activity of old antibiotics that have lost their effectiveness in treatment. It was previously reported that β-lactam antibiotics will restore the antibacterial activity against MRSA through the suppression of penicillin-binding protein 2a production, a key determinant for β-lactam antibiotic resistance, by chitosan-phytochemical conjugates [14]. Likewise, further study will determine the restored antibacterial activities with the detection of the 23S rRNA, the 16S rRNA, and *erm(X)* of *P. acnes* treated with CCA. This study highlights the limitations of current antimicrobial treatment strategies in patients with serious *P. acnes* infections.

## 4. Materials and Methods

### 4.1. Preparation of Chitosan-Phytochemical Conjugates

Chitosan produced from crab shell (*Chionoecetes japonicus*) chitin with a degree of deacetylation of 90% and average MW 310 kDa was provided from Kitto Life Co. (Seoul, Korea). Phytochemicals including caffeic acid, ferulic acid, and sinapic acid were purchased from Sigma-Aldrich Chemical Co. (St. Louis, MO, USA). All other chemicals and reagents used in this study were of analytical grade and commercially available. Chitosan-phytochemical conjugates were kindly provided by Prof. Jae-Young Je, Pukyong National University (Busan, Korea). The conjugates were prepared according to the previously reported method [13]. In brief, chitosan (0.25 g) was dissolved in 2% acetic acid (25 mL), and then 1.0 M hydrogen peroxide (0.5 mL) containing ascorbic acid (0.054 g) was added. Exactly 0.14 mM of phytochemicals (caffeic acid, ferulic acid, and sinapic acid) were added to the mixture after incubating at room temperature for 30 min, and then reacted for 24 h at room temperature. Results of the chitosan-phytochemical conjugates consisted of chitosan-caffeic acid conjugate (CCA), chitosan-ferulic acid conjugate (CFA), and chitosan-sinapic acid conjugate (CSA), respectively. A control (unmodified chitosan) was also treated with the same procedures without the addition of phytochemicals.

### 4.2. Bacterial Strains and Culture Conditions

The type bacterial strains were purchased from the Korean Collection for Type Cultures (KCTC; Daejeon, Korea); *P. acnes* KCTC 3314, *S. aureus* KCTC 1927, *S. epidermidis* KCTC 1370, and *P. aeruginosa* KCTC 1637. Two *P. acnes* clinical isolates were obtained from the Gyeongsang National University Hospital (Jinju, Korea), a member of the National Biobank of Korea. *P. acnes* strains were anaerobically cultured in brain heart infusion broth (Difco, Detroit, MI, USA) with 1.0% glucose supplement, and incubated in a CO_2_ incubator at 10% CO_2_-humidified atmosphere and 37 °C for 72 h (NAPCO 5400; General Laboratory Supply, Pasadena, TX, USA) in a 10% CO_2_-humidified atmosphere. Other bacteria were aerobically cultivated in Mueller–Hinton broth (MHB; Difco) at 37 °C. The broth dilution method was carried out in MHB according to the Clinical and Laboratory Standards Institute guidelines [47].

### 4.3. Determination of Minimum Inhibitory Concentration (MIC) and Fractional Inhibitory Concentration (FIC) Index

MIC was defined as the lowest concentration of crude extract that inhibited the visual growth after incubating the aerobic bacteria for 18 h and the anaerobic bacteria for 48 h. MIC values of the chitosan-phytochemical conjugate were determined by the two-fold serial dilution method in 96-well flat-bottomed microtitration plates at a final concentration of 7 × 10^5^ CFU/mL. The microtitration plates were read visually and the MIC of the chitosan-phytochemical conjugate that exhibited no turbidity was recorded as the MIC [5]. The interaction between chitosan-phytochemical conjugates and antibiotics including tetracycline, erythromycin, and lincomycin against acne-related bacteria was evaluated following the checkerboard method [48]. The synergy effect between chitosan-phytochemical conjugates and the antibiotics was tested as an FIC index [48]. Each FIC index was determined using the following equation:

∑FIC = FIC_A_ + FIC_B_ = C_A_/MIC_A_ + C_B_/MIC_B_

where MIC_A_ and MIC_B_ are the MICs of drugs A and B alone, respectively, and C_A_ and C_B_ are the concentrations of the drugs in combination, respectively. The synergistic effect was evaluated as an FIC index. The interaction was defined as synergistic if the FIC index was <1.0, additive if the FIC index was 1.0, subadditive if the FIC index was between 1.0 to <2.0, indifferent if the FIC index was 2, and antagonistic if the FIC index >2. Synergy was further sub-classified as marked (FIC index, <0.50) and weak (FIC index, between 0.50 to <1.0). 

## 5. Conclusions

In this study, we evaluated the antibacterial activity of chitosan-phytochemical conjugates against acne-related bacteria, such as *P. acnes*, *S. epidermidis*, *S. aureus*, and *P. aeruginosa*. Among the chitosan-phytochemical conjugates tested, CCA possessed significant antibacterial properties against acne-related bacteria. Furthermore, the combination of CCA with antibiotics used in the treatment of acne vulgaris resulted in a restoration of the effectiveness of commercial antibiotics used to treat skin pathogenic bacteria. Thus, the present study strongly suggested that chitosan-phytochemical conjugates such as CCA will be a good candidate for developing an alternative therapeutic agent with fewer side effects for the treatment of acne vulgaris.

## Figures and Tables

**Table 1:** Minimum inhibitory concentrations (MIC) of the chitosan-phytochemical conjugates against acne-related bacteria.

Strain	MIC (μg/mL)			
	CCA ^1^	CFA ^2^	CSA ^3^	Unmodified Chitosan
*Staphylococcus aureus* KCTC 1927	8	16	16	16
*Staphylococcus epidermidis* KCTC 1370	64	64	64	64
*Pseudomonas aeruginosa* KCTC 1637	16	32	32	32
*Propionibacterium acnes* KCTC 3314	256	256	256	512
*P. acnes* isolate 2874	256	256	256	512
*P. acnes* isolate 2875	128	256	256	256

^1^ CCA, chitosan-caffeic acid; ^2^ CFA, chitosan-ferulic acid; ^3^ CSA, chitosan-sinapic acid.

**Table 2:** Minimum inhibitory concentration (MIC) of tetracycline, erythromycin, and lincomycin against acne-related bacteria.

Strain	MIC (μg/mL)		
	Erythromycin	Lincomycin	Tetracycline
*Staphylococcus aureus KCTC 1927*	2	4	0.5
*Staphylococcus epidermidis* KCTC 1370	0.125	0.25	2
*Pseudomonas aeruginosa* KCTC 1637	16	64	0.125
*Propionibacterium acnes* KCTC 3314	1024	1024	32
*P. acnes* isolate 2874	1024	1024	16
*P. acnes* isolate 2875	0.125	1	0.125
Soussy’s MIC breakpoints ^1^	1–4 ^1^	2–8 ^1^	4–8 ^1^

^1^ Soussy et al. [32].

**Table 3:** Minimum inhibitory concentration (MIC) and fractional inhibitory concentration (FIC) indices of chitosan–caffeic acid (CCA) in combination with antibiotics against antibiotic-resistant acne-related bacteria.

Strains	Test Compound	MIC (μg/mL)	Median ∑FIC ^1^	∑FIC_max_^2^	∑FIC_min_^3^	Minimum Concentration for Observing Synergy
*P. acnes* KCTC 3314	CCA	256	0.533	1.016	0.188	64.0
	Tetracycline	32				4.0
*P. acnes* isolate 2874	CCA	256	0.502	1.004	0.375	64.0
	Tetracycline	16				4.0
*P. acnes* KCTC 3314	CCA	256	0.502	1.004	0.188	32.0
	Erythromycin	1024				64.0
*P. acnes* isolate 2874	CCA	256	0.502	1.016	0.313	64.0
	Erythromycin	1024				128.0
*Pseudomonas aeruginosa* KCTC 1637	CCA	16	0.5	1.063	0.266	4.0
	Erythromycin	16				0.5
*P. acnes* KCTC 3314	CCA	256	0.504	1.016	0.375	64.0
	Lincomycin	1024				256.0
*P. acnes* isolate 2874	CCA	256	0.502	1.016	0.313	64.0
	Lincomycin	1024				256.0
*P. aeruginosa* KCTC 1637	CCA	16	0.375	1.063	0.266	4.0
	Lincomycin	64				16.0

^1^ ∑FIC, the sum of FICs; ^2^ ∑FIC_max_, the highest, ∑FIC; ^3^ ∑FIC_min_, the lowest ∑FIC. The FIC index indicated synergistic effect: <0.5, marked synergy; 0.5 to <1.0, weak synergy; 1.0, additive; >1.0 to <2.0, subadditive; 2.0, indifferent; >2.0, antagonistic.
